# Qualitative Classification of Shear Wave Elastography for Differential Diagnosis Between Benign and Metastatic Axillary Lymph Nodes in Breast Cancer

**DOI:** 10.3389/fonc.2019.00533

**Published:** 2019-07-02

**Authors:** Shuyi Luo, Guangyu Yao, Zhe Hong, Shiyu Zhang, Weizhen Wang, Jingwen Zhang, Yaru Zhang, Junkai Wu, Li Zhang, Hong Cheng, Yi Hao, Yingjia Li

**Affiliations:** ^1^Department of Medicine Ultrasonics, Nanfang Hospital, Southern Medical University, Guangzhou, China; ^2^Breast Center, Nanfang Hospital, Southern Medical University, Guangzhou, China; ^3^Department of Imaging Diagnostics, Nanfang Hospital, Southern Medical University, Guangzhou, China; ^4^School of Biomedical Engineering, Southern Medical Uinversity, Guangzhou, China; ^5^Department of Ultrasound Diagnosis, Shenzhen Hospital, Southern Medical University, Shenzhen, China

**Keywords:** breast cancer, axilla, lymph node, elasticity imaging techniques, qualitative research

## Abstract

**Purpose:** To examine diagnostic performance of qualitative shear wave elastography (SWE) for evaluation of status of axillary lymph nodes (ALN) in comparison with conventional ultrasonograghy (US) and quantitative SWE parameters.

**Methods:** A total of 118 patients were enrolled, who were all scheduled for breast cancer surgery and core needle biopsy. Conventional US and SWE were performed before biopsy. Based on qualitative evaluation of each ALN, the SWE images were classified into four color patterns: Color Pattern 1: homogeneous; Color Pattern 2: filling defect within lymph node (LN); Color Pattern 3: homogeneous within LN with a localized colored area at the margin; and Color Pattern 4: filling defect within LN with a localized colored area at the margin. The diagnostic performances of the three methods were compared.

**Results:** There were 60 metastatic nodes and 61 benign nodes in the 121 ALNs. Benign ALNs were presented as Color Pattern 1 while metastatic ALNs usually were presented as Color Pattern 2 to 4 (*p* < 0.05). The AUC of qualitative SWE classification was 0.983, higher than that of quantitative SWE parameters and conventional US (*p*<0.05). The highest diagnostic performance, with AUC of 0.998, could be achieved if both conventional US and qualitative SWE were applied.

**Conclusion:** The qualitative SWE classification of ALNs proposed in our study exhibited better diagnostic performance than quantitative SWE parameters and conventional US, especially for differentiating metastatic ALNs from benign reactive ALNs. More accurate diagnosis could be reached with this new method and unnecessary biopsy might be avoided in the meantime.

## Introduction

The axillary lymph node (ALN) status is an important prognostic factor of breast cancer. At the same time, pre-operative ALN status provides significant reference for determining the clinical staging and treatment plan for breast cancer (1–3). Conventional US is a routine pre-operative evaluation of the ALN and its criteria for metastatic ALNs were based on morphologic characteristics, such as round shape, ratio of long axis to short axis (L/T) of <2, cortical thickening of more than 3 mm or absence of hilum (4), with resemblance to sonographic appearance of reactive ALNs.

As a non-invasive and reproducible technique in elastography, shear wave elastography (SWE) has been used in distinguishing benign from malignant lesions (5–8). Previous studies have revealed that SWE could differentiate between metastatic and benign lymph nodes in patients with thyroid nodules and suggested that Emax, Emean, and SD might be significant quantitative parameters for estimating cervical lymph nodes (9–11). As for the evaluation of SWE in ALNs in patients with breast cancer, only a few previous studies utilized quantitative SWE parameters for ALNs assessment (12–14). However, there is usually no shear wave signal within malignant lesions because malignant lesions with extremely low echo, particular hardness, or internal organization heterogeneity could lead to the inability to generate or propagate shear wave. Under this situation, the SWE image is not suitable to be measured by quantitative parameters. What's more, recent studies have demonstrated that the diagnostic performance of the combination of conventional US and qualitative classifications was higher than that of the combination of conventional US and quantitative parameters (*p* < 0.05), suggesting that qualitative SWE analysis might play an important role in clinical application (15). In clinical utility of SWE, we noticed that some of the ALNs showed high homogeneity, while some exhibited intra-nodal filling defect, and still some others displayed a localized colored area or even the “stiff rim” sign at the margin of lymph nodes, similar to the “black hole” and “stiff rim” sign in breast lesions (15). Therefore, it was worth exploring whether there was a link between the color elastic map and ALN status. Based on the qualitative SWE classification of breast lesions from the study of Cong (5), we proposed a qualitative classification method for diagnosis of ALNs.

In this study, we aimed to evaluate the diagnostic performance of qualitative SWE classifications for ALNs, in comparison with that of conventional US, quantitative SWE parameters and combinations of US and SWE parameters.

## Materials and Methods

### Patients

This prospective study was approved by Ethics Committee of Nanfang Hospital, Southern Medical University. And all patients who were enrolled had given their informed consent. Between April 2017 and December 2017, 158 patients with suspected breast cancer agreed to receive core needle biopsy of ALNs, SLNB (sentinel lymph node biopsy), and/or ALND (axillary lymph node dissection). They were examined by conventional US and SWE to evaluate ALNs status before biopsy. Among them, 118 females with a total of 121 ALNs were enrolled. One hundred fifteen patients had one ALN on the same side of breast cancer and the other three had two ALNs bilaterally. The mean age was 46.68 ± 10.07 years (age range, 27–69 years). The other 40 patients were excluded considering the following conditions: (i) patients who underwent neoadjuvant chemotherapy or radiotherapy before biopsy; (ii) patients whose ALNs were deeper than 30 mm (there would be a signal loss with elastography at this depth); (iii) patients with sub-standard SWE image (17 patients were excluded for this reason).

### US Examinations

All patients underwent conventional US and SWE by the Aixplorer ultrasound system (Supersonic Imagine, Aix-en-Provence, France) equipped with a linear SL4–15 MHz transducer. All examinations were performed by two doctors with 3–15 years of US operating experience on ALNs and 2 years in performing SWE. We collected the following lymph node morphological data: length of longest (L) and shortest (S) axes, L/S ratio, cortical thickness and presence or absence of hilum. After conventional US images were obtained, the maximum longitudinal plane of lymph node was selected and the ultrasound system was switched to the SWE penetration mode. Although SWE is reproducible, it could still be influenced by anatomic barriers of axilla and pre-compression artifacts (16). The pre-compression applied during the SWE examination could increase tissue stiffness. The transducer was lightly placed on the skin with a large amount of gel in order to obtain the SWE images of adequate quality and with fewest artifacts. The probe was kept still for a few seconds and the patients were instructed to hold breath for at least 5 s to let the elastography image stabilize, and meanwhile the color was constant in color elastic map for 3–5 s. Images without vertical stripe artifacts while presenting the surrounding fat as homogenous met the diagnostic standard. Subsequently, two images in longitudinal plane were recorded for each ALN. Only the most suspicious ALNs would be assessed by two radiologists. After independent assessment, they had a discussion and arrived at consensus if disagreement occurred.

### Evaluation of Conventional US

The scores of conventional US were calculated based on the 4 criteria: short-axis diameter (<7.0 mm, score 0; ≥7.0 mm, score 1), long-to-short-axis diameter ratio (<2.0, score 0; ≥2.0, score 1), hilum (present, score 0; absent, score 1), and cortical thickening (<3.0 mm, score 0; ≥3.0 mm, score 1) (4, 17).

### Evaluation of SWE Parameters

All measurements were performed along the longitudinal axis of ALNs to avoid an anisotropic phenomenon that different stiffness values could occur due to different measurements (12–14). The first 1-mm Q-Box (Q-Box1) was placed over the hardest intra- or peri-lesional area, and the second Q-Box (Q-Box2) of the same size was placed in the axillary fatty tissue. The following parameters were then determined: maximum stiffness (Emax, kPa), mean stiffness (Emean, kPa), minimum stiffness (Emin, kPa), ratio of lesion stiffness to that of the surrounding fat (Eratio), and SD (kPa).

**Figure 1:**
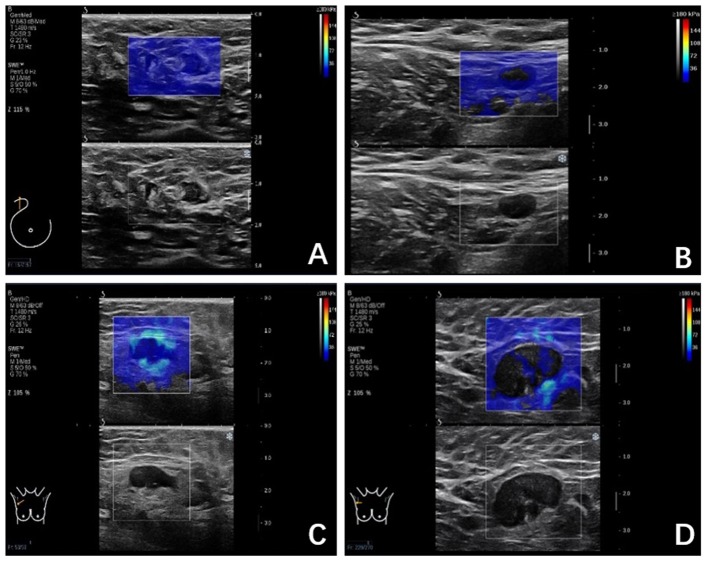
**(A)** Color Pattern 1, Homogeneous; Marginal sinus echo, homogeneous; Intra-lymph node echo, complete filling; **(B)** Color Pattern 2, Not very homogenous; Marginal sinus, homogeneous; Intra-lymph node echo, defect filling; **(C)** Color Pattern 3, Heterogeneous; Marginal sinus, heterogeneous; Intra-lymph node echo, complete filling; **(D)** Color Pattern 4. Heterogeneous; Margin echo, heterogeneous; Intra-lymph node echo, defect filling.

The SWE images of ALNs would be classified into four color patterns through the qualitative assessment, similar to a qualitative classification used in the diagnosis of breast lesions (5, 6): Color Pattern 1: homogeneous pattern (Figure 1A); Color Pattern 2: filling defect within lymph node (Figure 1B); Color Pattern 3: homogeneous within lymph node with a localized colored area at the margin (Figure 1C); Color Pattern 4: filling defect within lymph node with a localized colored area at the margin (Figure 1D). The classifications were based on the presence of “black hole” signs (6) or a localized colored area, manifested as increased stiffness in color elastic map.

After receiver operating characteristic (ROC) analyses, we obtained the optimal cutoff values of qualitative and quantitative SWE parameters to differentiate between metastatic and benign ALNs. When combining conventional US and SWE parameters, we made diagnosis in the following way. The ALNs were regarded as benign if the value of the SWE parameter was equal to or less than its optimal cutoff value, and those as metastatic ALNs if the SWE parameter was higher than its optimal cutoff value.

### Pathology

Patients included in our study all agreed to receive core needle biopsy of ALNs, SLNB, and/or ALND. All the Lymph nodes obtained after surgery were evaluated using a single H&E-stained section from each node. Metastases were defined as the presence of a tumor deposit > 0.2 mm in diameter in at least one lymph node. An experienced pathologist performed all histopathological analysis.

### Statistical Analyses

Statistical analyses were performed using SPSS, version 22.0 (SPSS, Chicago, IL, USA). Pathological results from biopsy were regarded as the reference standard. The chi-test was performed for categorical variables and an independent *t*-test was used for comparisons of continuous variables. The kappa statistics were calculated to assess the consistency between US (including conventional US, qualitative SWE and quantitative SWE) and pathological results.

The area under the ROC curves was obtained to compare the diagnostic performances of qualitative SWE, quantitative SWE, conventional US, and combination of conventional US and SWE. The *p* values <0.05 were considered as a statistical significance.

## Results

### Pathological Results

Of the 121 ALNs, 60 were metastatic and 61 were benign. ALN diameters ranged from 4.0 to 42.3 mm. The mean length of longest axis and shortest axis were 16.7 mm (6.2–42.3 mm) and 8.2 mm (4.0–25.0 mm). The mean size of metastatic ALNs was significantly larger than that of benign ALNs (*p* < 0.05).

### Diagnostic Performance of Conventional US Features

The average scores of benign and malignant lymph nodes by conventional US were 0.54 ± 0.79 points and 2.58 ± 0.87 points, respectively, with a significant difference (*p* < 0.05) (Table 1). The optimal cutoff value of this scoring standard was 1.5 points, with 0.938 AUC value, 91.7% sensitivity, 90.9% NPV, 82% specificity and 83.3% PPV (Table 2). Compared with pathology results, the kappa value of the scoring standard of conventional US was 0.736 (Table 2). The characteristics of misdiagnosed ALNs are summarized in Table 3.

**Table 1:** Shear wave elastography and conventional US parameters of 121 ALNs (Benign, *n* = 60, Metastatic, *n* = 61).

**Variable**	**Benign**	**Metastatic**	***p* value**
Conventional US	0.54 ± 0.79 points^*^	2.58 ± 0.87 points	*p* < 0.001
Emean	16.85 ± 6.44 kPa	49.93 ± 35.68 kPa	*p* < 0.001
Emin	14.82 ± 6.06 kPa	41.88 ± 32.67 kPa	*p* < 0.001
Emax	18.64 ± 7.08 kPa	54.79 ± 37.42 kPa	*p* < 0.001
SD	1.16 ± 0.93 kPa	3.74 ± 3.16 kPa	*p* < 0.001
Eratio	1.55 ± 0.62	5.65 ± 4.84	*p* < 0.001
**QUALITATIVE SWE**			
1	61 (100%)	2 (3.3%)	*p* < 0.001
2	0	7 (11.7%)	
3	0	22 (36.7%)	
4	0	29 (48.3%)	

* *Values are presented as the mean ± standard deviation or number (%)*.

**Table 2:** Diagnostic performances of conventional US, shear wave elastography (SWE) parameters, and combination of conventional US and SWE parameters.

**Parameters**	**Cutoff value**	**AUC**	**Sensitivity (%)**	**Specificity (%)**	**Positive predictive value (PPV) (%)**	**Negative predictive value (NPV) (%)**	**Aocuracy**	**Kappa value^***c***^**
Conventional US	1.5	0.938	91.7	82	83.3	90.9	86.8	0.736
Emax (kPa)	26.05	0.944	93.3	88.5	88.9	93.1	90.9	0.818
Emean (kPa)	26.90	0.946	86.7	96.7	96.3	88.1	91.7	0.835
Emin (kPa)	22.75	0.910	81.7	90.2	89.1	83.3	86.0	0.719
Eratio	1.85	0.930	90	852	87.7	89.7	87.6	0.752
SD (kPa)	2.05	0.833	70	91.8	89.4	75.7	81.0	0.619
Qualitative	2	0.983	96.7	100	100	96.8	98.3	0.967
**SWE**								
Combination 1^a^	NA	0976	95	95.1	95	95.1	95	0.901
Combination 2^b^	NA	0.998	98.3	100	100	98.4	99.2	0.983

a *Combination 1, combination of conventional US and quantitative SWE parameters*.

b *Combination 2, combination of conventional US and qualitative SWE classification*.

c *kappa value of different assessment compared with pathology, a kappa in the range of 0.21–0.40 be considered “fair” agreement kappa = 0.41-0.60 be considered “moderate” agreement, kappa = 0.61-0.80 be considered “substantial” agreement, kappa > 0.81 be considered “almost perfect” agreement*.

**Table 3:** Misdiagnosed lymph nodes according to conventional US and qualitative SWE classification.

**Case**	**Color pattern**	**Age**	**Size (mm)**	**Emax (kPa)**	**Emean (kPa)**	**Emin (kPa)**	**Esd (kPa)**	**Eratio**	**Conventional US**	**Pathology**
**FALSE-POSITIVE LYMPH NODES ACCORDING TO CONVENTIONAL US**										
1	1	46	7.5	24.4	20.6	16.9	2.3	1.5	2	Benign
2	1	32	20.0	25.9	24.9	23.5	0.8	1.0	2	Benign
3	1	32	8.0	11.7	10.6	9.4	0.8	1.5	3	Benign
4	1	39	35.0	24.5	23.7	22.6	0.6	1.8	2	Benign
5	1	36	27.0	25.8	24.9	23.6	0.9	2.2	2	Benign
6	1	48	8.4	23.9	19.5	11.7	3.1	1.2	2	Benign
7	1	50	9.0	22.2	19.3	16.7	1.6	1.7	2	Benign
8	1	45	1.0	8.1	7.6	7.1	0.3	4.7	2	Benign
9	1	39	33.0	22.5	21.0	17.5	1.4	1.1	2	Benign
**FALSE-NEGATIVE LYMPH NODES ACCORDING TO CONVENTIONAL US**										
1	3	45	15.2	20.3	19.6	18.8	0.5	1.1	0	Metastasis
2	3	44	15.8	32.6	27.9	22.8	2.7	5.1	1	Metastasis
3	3	45	15.7	26.4	25.5	23.9	0.9	2.1	1	Metastasis
4	2	52	16.0	26.3	17.7	12.8	4.0	1.1	1	Metastasis
5	3	67	17.0	35.3	34.3	32.5	1.0	2.9	1	Metastasis
**FALSE-NEGATIVE LYMPH NODES ACCORDING TO QUALITATIVE SWE CLASSIFICATION**										
1	1	48	19.0	13.6	12.3	10.3	1.2	1.9	2	Metastasis
2	4	45	26.0	12.5	12.2	11.5	0.3	1.6	2	Metastasis

### Diagnostic Performance of Quantitative SWE Features

The mean value of Emean, Emin, Emax, SD, and Eratio was significantly higher in metastasis lymph nodes than in benign lymph nodes (Table 1): Emean, 49.93 ± 35.68 vs. 16.85 ± 6.44 (kPa); Emin, 41.88 ± 32.67 vs. 14.82 ± 6.06 (kPa); Emax, 54.79 ± 37.42 vs. 18.64 ± 7.08 (kPa); SD, 3.74 ± 3.16 vs. 1.16 ± 0.93 (kPa); and Eratio, 5.65 ± 4.84 vs. 1.55 ± 0.93 (all, *p* < 0.05). The optimal cutoff value of Emean, Emin, Emax, SD and Eratio were 26.90, 22.75, 26.05, 2.05 and 1.85 kPa, the corresponding kappa value were 0.835, 0.719, 0.818, 0.619, and 0.752, respectively. The AUC values of quantitative SWE parameters were summarized in Table 2. Among all the quantitative SWE parameters, the AUC value of Emean was the highest.

### Diagnostic Performance of Qualitative SWE Features

**Figure 2:**
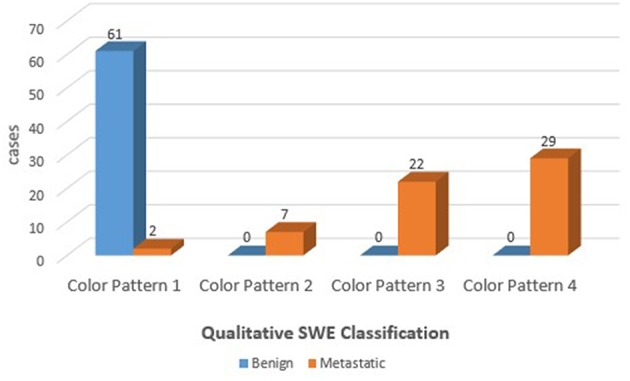
Pathological results of qualitative classification of ALNs.

**Figure 3:**
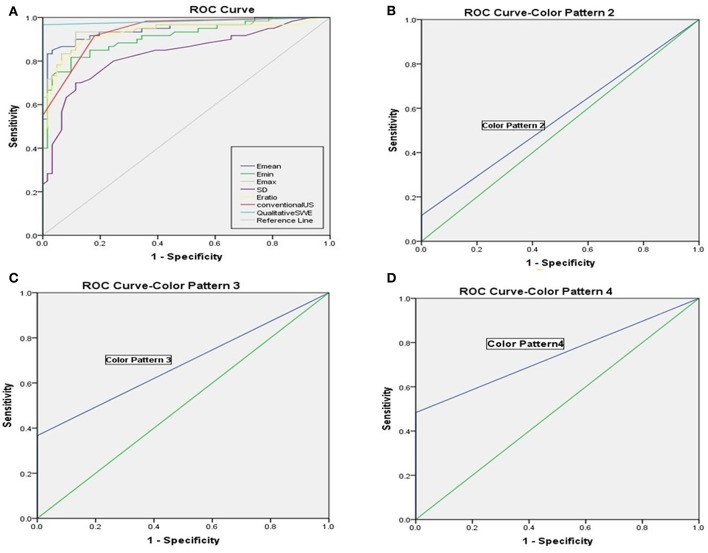
**(A)** Receiver operating characteristic curves of qualitative SWE classification pattern, quantitative SWE parameters and conventional US. **(B)** Receiver operating characteristic curves of Color Pattern 2 with the AUC value was 0.558. **(C)** Receiver operating characteristic curves of Color Pattern 3 with the AUC value was 0.683. **(D)** Receiver operating characteristic curves of Color Pattern 4 with the AUC value was 0.742.

The benign ALNs presented as Color Pattern 1, while metastatic ALNs usually presented as Color Pattern 2 to 4 (Figure 2). There were significant differences in qualitative SWE patterns between metastatic and benign ALNs (*p* < 0.05) (Table 1). The AUC value of qualitative SWE patterns was 0.983, significantly higher than that of conventional US (*p* < 0.05) and all quantitative SWE parameters (*p* < 0.05) (Figure 3A). The optimal cutoff value of qualitative SWE as a diagnostic standard of metastatic lymph nodes was Color Pattern 2, with 96.7% sensitivity, 100% specificity, 96.8% NPV and 100% PPV (Table 2). Compared with pathology, the kappa value of qualitative SWE assessment for lymph node was 0.967 (Table 2).

Further analysis on the results above showed that when regarding Color Pattern 2 (Figure 3B), Color Pattern 3 (Figure 3C), and Color Pattern 4 (Figure 3D) as diagnostic standard of metastatic ALNs, respectively, the diagnostic performance of Color Pattern 4 (AUC = 0.742) was higher than Color Pattern 2 (AUC = 0.558) and 3 (AUC = 0.683), which was consistent with the common SWE performance of metastatic ALNs in clinic.

**Figure 4:**
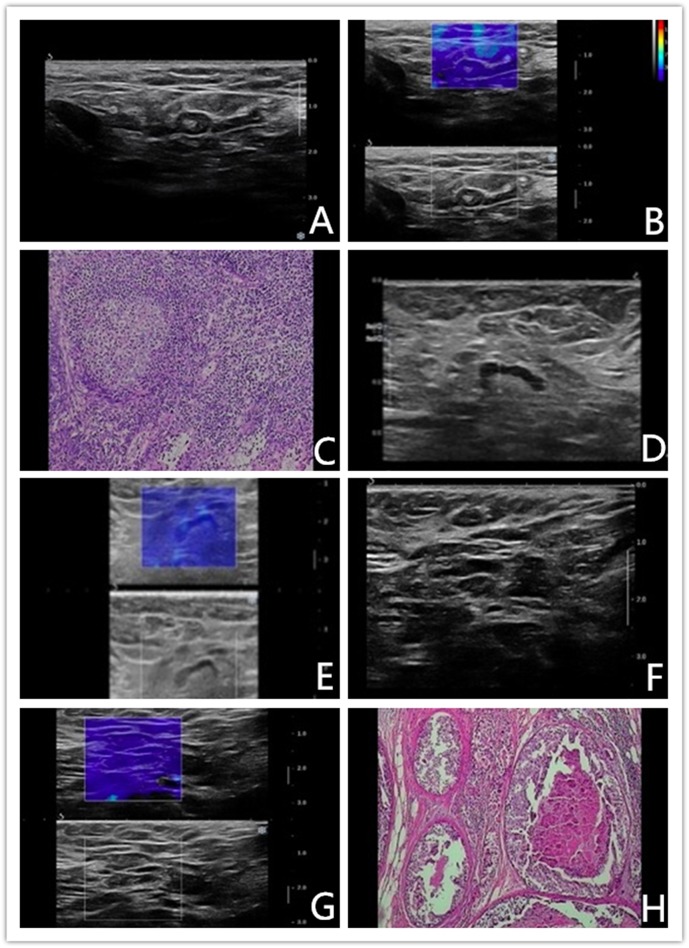
**(A–C)** Benign axillary lymph node in a 46-years-old woman. Conventional US revealed an elliptical-shaped, sharp-bordered lymph node with cortical thickness of 3.5 mm, the diameter of short axis of 4.2 mm and L/S ratio <2, which was diagnosed as metastatic ALN. Shear wave elastography revealed qualitative classification of Color Pattern 1 for this ALN, diagnosed as benign ALN. Reactive hyperplasia of lymphocyte and histocyte within the axillary lymph node. **(D–E)** Malignant axillary lymph node in a 67-years-old woman. Conventional US revealed an elliptical-shaped lymph node with cortical thickness of 2.5 mm, the diameter of short axis of 7 mm and L/S ratio >2, which was diagnosed as benign ALN. Shear wave elastography revealed qualitative classification of Color Pattern 3 for this ALN, diagnosed as metastatic ALN. **(F–H)** Micrometastatic axillary lymph node in a 48-years-old woman. Conventional US revealed an elliptical-shaped lymph node with cortical thickness of 2.7 mm, the diameter of short axis of 7 mm and L/S ratio >2, which was diagnosed as benign ALN. Shear wave elastography revealed qualitative classification of Color Pattern 1 for this ALN, diagnosed as benign ALN. Pathological exam diagnosed as micrometastasis.

If Color Pattern 2 was used as a diagnostic threshold, nine benign reactive ALNs presented as Color Pattern 1 were diagnosed as benign lymph nodes by qualitative SWE classification, while all of them were misdiagnosed as metastatic lymph nodes by conventional US (Figures 4A–C). Discerning reactive lymph nodes from metastatic ALNs by qualitative SWE patterns, we achieved a lower false positive rate of qualitative SWE classification (0%) than that of conventional US (18%). Five of 60 metastatic ALNs were misdiagnosed as benign by conventional US. In contrast, they were highly suspected as metastatic lymph nodes (Figures 4D,E) because they were ranged as Color Patterns 2–3 by qualitative SWE patterns. The false negative rate of qualitative SWE classification was lower than that of conventional US: 3 vs. 8%. Furthermore, two of the 60 metastatic ALNs in this study were actually micrometastases, but they were regarded as benign by conventional US, qualitative and quantitative SWE (Figures 4F–H). The characteristics of misdiagnosed ALNs in qualitative SWE classification were summarized in Table 3.

**Figure 5:**
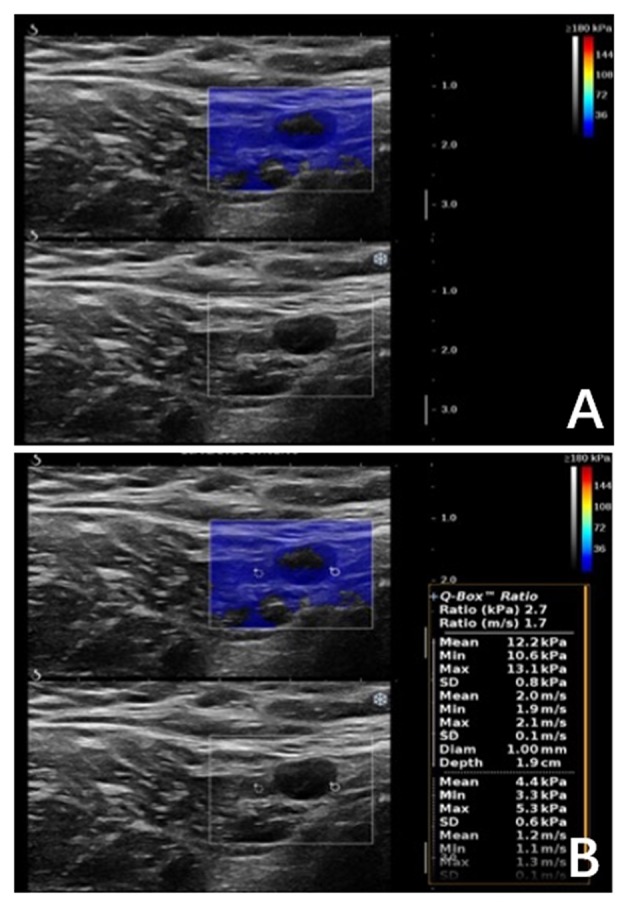
Malignant axillary lymph node in a 63-years-old woman. **(A)** Shear wave elastography revealed qualitative classification of Color Pattern 2 for this ALN, diagnosed as metastatic ALN. **(B)** All the SWE parameters of the ALN were under cut-off threshold, which was diagnosed as benign ALN.

In our study, a portion of metastatic ALNs presented as Color Pattern 2 were highly suspected as metastatic ALNs, while all the quantitative SWE parameters under the diagnostic threshold were misdiagnosed as benign ALNs (Figures 5A,B). In these cases, ALN status could not be accurately assessed by SWE quantitative parameters.

### Diagnostic Performance of Combining SWE Parameters With Conventional US

The diagnostic performance of combining SWE parameters and conventional US was summarized in Table 2. The AUC value of the combination of conventional US and qualitative SWE classification was 0.998, higher than that of the combination of conventional US and Emean (0.976), and conventional US (0.938), qualitative SWE classification (0.983), and Emean (0.946) alone. Moreover, the combination of conventional US and qualitative SWE classification obtained a significantly higher specificity (100%) than that of conventional US (82%).

## Discussion

Most of the previous studies focusing on strain elastography as a preoperative evaluation of ALNs have revealed that higher stiffness presented as blue in the color elastic map was positively correlated with higher strain ratios in metastatic ALNs than benign lymph nodes (18). However, strain elastography could only provide qualitative information with poor reproducibility and high operator dependence. Conversely, SWE is reproducible (19) and can provide qualitative and quantitative information of tissue elasticity. Nevertheless, not all patients with breast cancer could achieve SWE images of ALNs meeting the diagnostic standard (images without vertical stripe artifacts while presenting the surrounding fat as homogenous) due to the anatomic features of axilla. And SWE images standardization is significant for accurate diagnose. In our study, all the patients with standard SWE images of ALN were included and 17 patients with sub-standard SWE images were excluded, though all of them have received standard SWE operation.

Current studies (12, 14) have confirmed the significance of quantitative SWE parameters in evaluation of axillary lymph node status. In our study, the diagnostic performances of qualitative SWE and some of the quantitative SWE parameters were higher than that of conventional US. And all SWE parameters including qualitative SWE patterns and quantitative SWE parameters were statistically significantly different between metastatic and benign ALNs. The optimal cutoff value of Emean, Emin, Emax, and Eratio in our study was different from those in previous studies. The reason might be that Shear-wave elasticity values vary due to different ROI settings (20). In order to reflect the elasticity for the hardest area of the ALN and reduce the containing of surrounding fatty tissue as much as possible, the Q-BOX diameter was set at 1 mm in our study smaller than that of the previous studies (12, 14). So far as we know, previous studies suggested that quantitative SWE parameters with different cutoff values could exhibit different diagnostic performance (12, 14), and quantitative SWE parameters as indicators for metastatic ALNs were not standardized. We found that under the conditions of images with adequate quality, a portion of metastatic lymph nodes presented as filling defect in color elastic map suggested metastatic ALNs, but they were misdiagnosed as benign ALNs considering the quantitative parameters of them were still below the optimal cutoff values. In these cases, quantitative parameters might not be used to assess ALNs status properly.

Here, we classified the ALN SWE images visually into four patterns. The benign ALNs presented as Color Pattern 1 (homogeneous in color elastic map), while metastatic ALNs usually presented as Color Pattern 2 to 4 (filling defect or a localized colored area at the margin). Although previous studies have demonstrated “black hole” sign and “stiff rim” sign (15) in SWE images of breast cancer, no particular description has been reported in ALNs. Cancer cells usually invade the edge of the ALNs through the afferent lymphatic vessel at early metastasis. And the increasing density of tumor cells would result in elasticity changes and a localized colored area at the marginal sinus. Thereafter, the cells would spread to the medullary sinus and the whole lymph node, leading to the significant increase of lymph node stiffness. Gradually, cancer cells dominate the lymph node and infiltrate the envelope leading to adhesion to the surrounding tissues and hyperplasia of surrounding interstitial fibrous tissues. Finally, they might present as “stiff rim” at the margin of metastatic ALNs. As for the presence of filling defect within the metastatic lymph, there are three possible explanations. The first is that low shear wave amplitude within the metastatic lymph nodes was caused by attenuation of the energy of the shear wave at the margin of metastatic lymph node (21). The second is that metastatic ALN with extremely low echo or hardness exceeded the threshold value of SWE, resulting in the inability to produce, amplify, propagate or measure shear waves (6). The third reason might be that the growth characteristics of cancer cells within the metastatic ALNs, including heterogeneity of cancer cells, formation of new vessels, necrosis and inflammation, might block the generating, transmitting, and/or testing of shear waves (22).

In our study, we obtained better diagnostic performances with the proposed qualitative SWE classification than the quantitative SWE parameters or conventional US (*p* < 0.05). According to the qualitative classification, the color patterns of metastatic ALNs were statistically different from that of benign ALNs. This was consistent with previous studies of qualitative SWE evaluation of breast lesions (5–7). Meanwhile, based on results of our study, qualitative SWE pattern for ALNs not only simplified clinical work, but also increased diagnostic accuracy, with sensitivity up to 96.7% and specificity 100%, mainly owing to benign ALNs presented as Color Pattern 1. Furthermore, the combination of qualitative SWE patterns and conventional US had a higher AUC value than that of conventional US alone or combination of conventional US and any other quantitative parameters (Table 2).

We have found that some ALNs were misdiagnosed by conventional US or qualitative SWE classification (Table 3). Two of the 60 metastatic ALNs in this study were micrometastases. Specifically, the ones with diameter of a single biggest metastasis ranging from 0.2 to 2.0 mm or the number of tumor cells in a biggest metastasis >200 were all defined as benign by conventional US, qualitative and quantitative SWE (Figures 4F–H). The reason might be that the invasion region was too small to be discovered by current examinations. There were five ALNs highly suspected as metastasis lymph nodes and misdiagnosed as benign by conventional US, but actually they belonged to Color Patterns 2–3 (Figures 4D,E). In early ALNs metastasis, due to the close arrangement of tumor cells, part of the lymph node marginal sinuses are stiffer than the area within lymph nodes, with no significant morphological changes. This infiltrating characteristic prevents conventional US from detecting early stage lymph node metastasis, which might be discerned by qualitative SWE, presenting as Color Pattern 2–3 in color map. There were nine reactive ALNs misdiagnosed as metastatic lymph nodes by conventional US because of the cortical thickening of >3 mm and L/T ratio of <2. All of them, however, were diagnosed as Color Pattern 1 by qualitative SWE classification, namely, they were benign lymph nodes (Figures 4A–C). Reactive lymph nodes usually manifest as lymphadenectasis and cortical thickening, similar to the metastatic lymph nodes in conventional US images. But the fact is that there are no elasticity changes in neither peri- nor intra-lymph nodes. Consequently, they were classified into Color Pattern 1 by qualitative SWE classification. Therefore, the combination of conventional US and qualitative SWE classification could improve the diagnostic accuracy of benign reactive lymph nodes and avoid unnecessary biopsy for the patients in question. Also, qualitative SWE classification might be able to increase the positive rate of ALN biopsy given that this method could be applied to guide puncture biopsy.

Nonetheless, the study has some limitations. First, in order to ensure the node-to-node correlation between SWE and pathology, only the patients who had agreed to receive core needle biopsy and/or surgical biopsy for ALNs were included. Hence, SWE was not performed consecutively on all patients. Second, the sample size was small and there should be further studies with larger samples to validate our findings.

In conclusion, the qualitative SWE classification of ALNs proposed in our study exhibited better diagnostic performance than quantitative SWE parameters and conventional US, especially for differentiating diagnosis of metastatic ALNs from benign reactive ALNs. By discerning the elastic changes of ALNs, the qualitative SWE classification method owns the advantage of more accurate diagnosis. Moreover, it could avoid unnecessary biopsy and might guide for accurate ALN puncture biopsy.

## Ethics Statement

This study was carried out in accordance with the recommendations of Ethics Committee of Nanfang Hospital, Southern Medical University with written informed consent from all subjects. All subjects gave written informed consent in accordance with the Declaration of Helsinki. The protocol was approved by the Ethics Committee of Nanfang Hospital, Southern Medical University.

## Author Contributions

YL, SL, GY, HC, and YH read the literature and determine research topic. SL, ZH, SZ, WW, and YZ collecting data. JW and LZ statistics. JZ modify the article.

### Conflict of Interest Statement

The authors declare that the research was conducted in the absence of any commercial or financial relationships that could be construed as a potential conflict of interest.

## Abbreviations

ALN: Axillary lymph node
SWE: Shear wave elastography
US: Ultrasonography
AUC: Area under the curve.
